# Cross-Dataset Evaluation of Deep Learning Networks for Uterine Cervix Segmentation

**DOI:** 10.3390/diagnostics10010044

**Published:** 2020-01-14

**Authors:** Peng Guo, Zhiyun Xue, L. Rodney Long, Sameer Antani

**Affiliations:** Communications Engineering Branch, Lister Hill National Center for Biomedical Communications, U.S National Library of Medicine, National Institutes of Health, Bethesda, MD 20894, USA; zhiyun.xue@nih.gov (Z.X.); rlong@mail.nih.gov (L.R.L.); santani@mail.nih.gov (S.A.)

**Keywords:** deep learning, uterine cervical cancer, uterine cervix segmentation, automated visual evaluation, Mask R-CNN, Mask^X^ R-CNN

## Abstract

Evidence from recent research shows that automatic visual evaluation (AVE) of photographic images of the uterine cervix using deep learning-based algorithms presents a viable solution for improving cervical cancer screening by visual inspection with acetic acid (VIA). However, a significant performance determinant in AVE is the photographic image quality. While this includes image sharpness and focus, an important criterion is the localization of the cervix region. Deep learning networks have been successfully applied for object localization and segmentation in images, providing impetus for studying their use for fine contour segmentation of the cervix. In this paper, we present an evaluation of two state-of-the-art deep learning-based object localization and segmentation methods, viz., Mask R-convolutional neural network (CNN) and Mask^X^ R-CNN, for automatic cervix segmentation using three datasets. We carried out extensive experimental tests and algorithm comparisons on each individual dataset and across datasets, and achieved performance either notably higher than, or comparable to, that reported in the literature. The highest Dice and intersection-over-union (IoU) scores that we obtained using Mask R-CNN were 0.947 and 0.901, respectively.

## 1. Introduction

According to the World Health Organization (WHO), there were about 570,000 new cases of invasive cervical cancer diagnosed in 2018, representing almost 7% of all female cancers [1]. Early detection and treatment of cervical cancer/pre-cancer can improve survival rate. Various screening modalities include the Pap test, Visual Inspection with Acetic acid (VIA), and Human Papillomavirus (HPV) type detection. The VIA test is conducted by applying dilute (3–5%) acetic acid to the cervix during a gynecological exam, which causes temporary whitening of HPV infected tissue, and then visually inspecting the cervix [2]. However, while the VIA is simple and inexpensive, high subjectivity has been reported in [3] where an agreement rate of only 56.8% (538/948 cases) was obtained from 20 expert colposcopists (12 general gynecologists and 8 gynecologist-oncologists).

Recently, a deep learning-based method called Automatic Visual Evaluation (AVE) was shown to have the potential to replace VIA as an automatic screening application [4]. The method shows promise for use in low-to-medium resource settings with high disease burden particularly for pre-menopausal women in the age group (25–49) who tend to exhibit higher incidence of visually detectable cervical pre-cancer. The algorithm utilizes an object detection network which is trained using images from a longitudinal cervical cancer screening study in Costa Rica. The training of the network requires bounding boxes surrounding the cervix in the images. While bounding boxes are convenient for AVE, they are coarse annotations that may contain a significant amount of irrelevant information, such as clinical instruments (the speculum and swab) and other non-cervical tissues (vaginal walls). In AVE, the impact of these artifacts is offset by the large number of images used in the study. However, for tracking lesions on cervical tissue for potential future use with automated diagnostic or therapeutic recommendations, it would be desirable to develop a high-quality automatic cervix segmentation technique. Further, accurate cervix boundary extraction is necessary for determining the cervix type. Also, detecting the squamo-columnar junction or the transformation zone (T-zone) [5,6] is aided by this process. In this paper, we evaluate the use of recent deep learning-based techniques to automatically localize and segment the cervix region.

**Figure 1:**
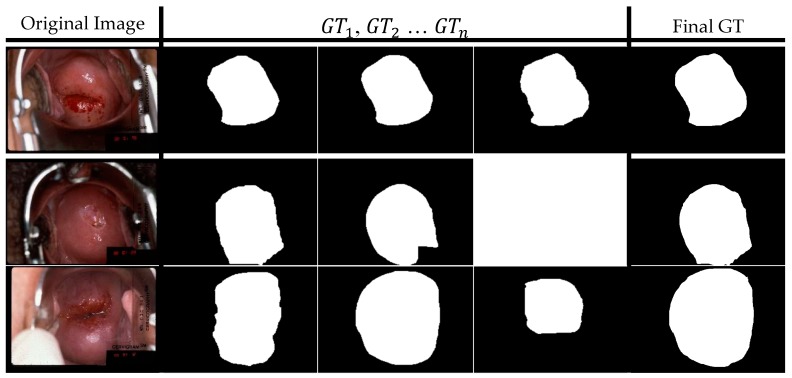
Atypical Squamous Cells of Undetermined Significance/Low-grade Squamous Intraepithelial Lesion (ASCUS/LSIL) Triage Study (ALTS) dataset image examples (Original Image), the samples of experts’ annotations ($GT_{1}$–$GT_{n}$ ) and the final ground truth (Final GT), the white blobs denote the cervix region. Note that the 2nd row image is labeled by 2 experts with only 2 annotation masks, so the 3rd column is blank. The 1st and 3rd row images have 3 or more than 3 (not shown) annotation masks. The “Final GT” is decided as the union of all the available masks of that image.

In order to improve and make a segmentation algorithm robust, it is necessary to train it on a large number of images acquired using a variety of devices (e.g., digital and film camera and smartphone) from different geographic regions. In this work, we use three datasets collected in different geographical regions using different data acquisition devices. The datasets have large intra- and inter-dataset variability with respect to object illumination, lighting conditions, object color, object position, size, and other related visual factors. Our observation of the images in these datasets is that while they capture the natural variety in cervix appearance, they are subject to wide variations in image quality [7]. Image quality is a combination of the coverage of the anatomical region of interest, focus, illumination, reduced specular reflection off the cervix, absence/reduced presence of clinical instruments, such as the speculum and swab, and variety in camera zoom levels. Also, a large fraction of the images in the datasets are weakly annotated with bounding boxes only. Of the small fraction of images that are strongly annotated with segmentation masks, almost half of them are labeled by more than one expert for each image. This introduces the need to manage multiple “ground truths” due to inter-observer variability among the experts annotating the images (Figure 1) [8].

Our goal is to investigate state-of-the-art deep learning methods for automatic cervix region segmentation that are resilient to variation in expert annotation and robust across diversity in cervix appearance. Toward this end, we evaluate the algorithms on multiple strongly and weakly annotated datasets to demonstrate not only the robustness, but also the advantage in using weakly annotated images as a part of the training set instead of just employing a large number of strongly annotated images.

Main contributions of this work include: (1) comparing two state-of-the-art deep learning segmentation algorithms, namely, Mask R-convolutional neural network (CNN) [9] and Mask^X^ R-CNN [10] for cervix segmentation through (2) extensive single and multi-dataset evaluation using three large cervix image datasets; and, (3) demonstrating improvement through higher Dice metric and intersection-over-union (IoU) scores on the same dataset(s) used in prior studies. The rest of the paper is organized as follows: Section 2 reviews the related studies that have been reported in the literature, Section 3 describes the two deep learning architectures that we use in this work; Section 4 and Section 5 present the experiments, the results and the discussion; Section 6 concludes the study in this paper.

## 2. Related Work

We broadly divide prior work done in segmenting cervix regions in photographic cervical images into two categories: (1) those that use conventional image processing techniques, and may use traditional machine learning approaches; and, (2) those that apply deep learning approaches.

### 2.1. Conventional Image Processing Techniques

Conventional image processing techniques require human expertise in selecting and extracting visual (or pixel-based) features that separate the cervix region from non-cervix regions. They are hampered by: (i) variability in image appearance (e.g., presence of blood, specular reflection from the moistened tissue, texture variation due to pathology, and occlusion due to medical instruments); and, (ii) feature selection and representation for maximizing discrimination between the cervix region from non-cervix regions. In [11], K-means clustering and Otsu’s method are applied to extract the cervix region after the step of specular reflection removal, by replacing the region with patches of similar texture and color. The segmentation is based on thresholding pixel values in HSV and LAB color space, which might not be robust across data sets when either color or lighting condition changes. An IoU score of 0.79 is reported in [11]. In [12], cervix segmentation serves as the first step for the cervix image registration workflow; similar to [11], a fuzzy C-means clustering approach is employed for cervix segmentation and is reported to achieve an average IoU score of 0.76. In [13], cervix images captured using cell-phone cameras are segmented through the combination of edge detector, red-color component filter, curvature filter, and thresholding. The researchers report an average Dice score of 0.9, on a small set of 151 images. Curvature features are used in [14,15] with additional information provided by shape priors, the proposed curve evolution with the shape prior model achieves an average Dice measure of 0.81. In addition to the above image processing techniques, statistical modeling methods have also been employed for cervix segmentation in the literature. In [16], the probability function is defined by adding a circular/elliptical prior to the existing framework with K-means clusters from [14]. The model is shape-based in which the cervix can be considered either as circular or elliptical, and the reported average Dice score is 0.75. In [17], the probability of a pixel being part of cervix is calculated by maximizing the likelihood function. The performance measure used in [17] is the ROC (receiver operating characteristic) curve which has an AUC (area under curve) with approximate value 0.9 and is obtained from 250 sample images acquired from only 4 patients. Sparse representation and group sparsity based feature selection are employed in [18,19,20] to segment the biomarker of acetowhitened [6] area on cervix, and a sensitivity improvement of 0.12 and specificity of 0.05 is achieved [19] using discriminative sparse representation compared with the sparse representation approach used in [20].

### 2.2. Deep Learning-Based Techniques

Although deep learning techniques have been widely applied for segmentation tasks in various medical image modalities, few research groups report applying them for cervix region segmentation in photographic images of the uterine cervix. The deep learning U-Net [21] algorithm was applied for the work reported in [22] on 6692 images from the Intel & MobileODT Cervical Cancer Screening Competition [6]; these images were cropped into 32 × 32 pixel patches; the quantitative results were not reported, however, this might be because, similar to [17], the cervix segmentation was just used as preprocessing for the subsequent classification network. In [23,24,25], a convolutional neural network (CNN) model is implemented over a cervix dataset collected by MobileODT (Kaggle Dataset); the reported average Dice score is 0.67. Very recently, one research group applied Mask R-CNN on cervix segmentation tasks, the obtained (Dice, IoU) score is (0.8711, 0.765) on “Kaggle Dataset” as reported in [26]. We use the same dataset in our study and compare our results with these techniques.

## 3. Data

Three datasets were used in this evaluation. They are the Costa Rica Vaccine Trial (CVT) dataset, the Atypical Squamous Cells of Undetermined Significance/Low-grade Squamous Intraepithelial Lesion (ASCUS/LSIL) Triage Study (ALTS) dataset, both collected by the National Cancer Institute (NCI), and the “Kaggle Dataset” which is from the Intel & MobileODT Cervical Cancer Screening Competition hosted on Kaggle.

### 3.1. CVT Dataset

The Costa Rica Vaccine Trial (CVT) was conducted by the NCI and its partners in agreement with the Ministry of Health of Costa Rica [27,28]. After receiving the HPV16/18 vaccines, women participating in the study were referred to colposcopy if the cervical lesion persisted or if they had high grade cytology at any time during the trial. The images of the uterine cervix of women referred to colposcopy were acquired (with permission obtained during enrollment in the study) on photographic film and digitized. The dataset, which we call set A, consists of 3398 images (from 1674 unique patients) that we split into 80%/20% training and testing partitions. The split is performed randomly on the patient level to ensure that no woman’s images are in both training and testing. This prevents data leakage that might be brought about by using multiple images captured from the same woman in both the training and test sets. All the images in this dataset have cervix segmentation masks created by an expert gynecologist. From this CVT set we derived our two subsets: (i) $A_{mask}$ which refers to the subset of images that have the fine segmentation masks; and, (ii) $A_{box}$ which refers to the subset of images that only have coarse bounding box surrounding the cervix.

### 3.2. ALTS Dataset

As reported in [29], the atypical squamous cells of undetermined significance/low-grade squamous intraepithelial lesion (ASCUS/LSIL) Triage Study (ALTS), also conducted by the NCI, was a randomized clinical trial conducted in the United States that was designed to determine the optimal management plan for low-grade cervical abnormalities. The ALTS image data used in our work, that we call set B, is a subset of 939 images taken from the ALTS study for the purpose of studying observer variability in visual interpretation of cervical abnormalities. These images were annotated using the Boundary Marking Tool (BMT) developed by the National Library of Medicine (NLM) by several medical experts in cervical oncology. To resolve small variations in the boundary markings from multiple expert observers, our naïve approach was to define the union of these individual boundaries as the final ground truth (GT) for each image (Figure 1). Next, the bounding box annotations are created by plotting a rectangular box tightly enclosing the obtained union. One should only use dataset B entirely for training or testing, but not for both since the patient ID associated with each image is not available and randomly splitting the dataset would result in data leakage. From this dataset we derived our two subsets $B_{mask}$ and $B_{box}$.

### 3.3. Kaggle Dataset

**Figure 2:**
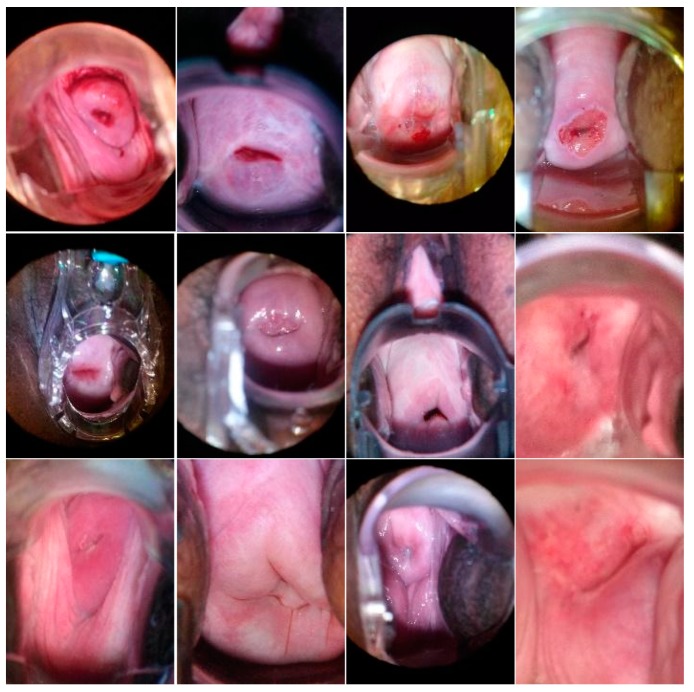
Example images from Kaggle [6] showing cervix images of different types. Type 1 is on the top row, while Types 2 and 3 are on the middle and bottom rows, respectively.

This dataset containing 1448 training images and 512 testing images is collected by MobileODT using their digital cervical imaging device for Enhanced Visual Assessment (EVA). It is part of the data hosted on Kaggle [6] for the “Intel & MobileODT Cervical Cancer Screening Competition” whose goal is to develop novel algorithms that classify the images into the three cervix types related to the age progression in women (Figure 2). The image annotations include cervix region contours which were made available by Fernandes [25]. The quality of these images varies widely, and includes motion blur, presence of the speculum and its reflection on the cervix tissue, poor lighting, poor camera positioning, among other flaws. From this “Kaggle dataset” we derived our two subsets $C_{mask}$ and $C_{box}$.

For the testing split of Datasets A and C, no bounding box annotation is generated since bounding boxes for the tested images are not needed in evaluating the segmentation performance. We denote the available mask/bounding box annotations as “*mask*” or “*box*”. “*mask*” refers to the mask annotations generated for the training split of the dataset(s). We summarize in Table 1 the quantitative details of the three datasets used:

## 4. Methods

As mentioned earlier, we investigate two state-of-the-art deep learning architectures, viz., Mask R-CNN and Mask^X^ R-CNN, for the cervix segmentation problem. We categorize our datasets into strongly or weakly labeled subsets (*mask* and *box*). Strongly labeled sets have fine cervix boundaries from which we derive masks of the cervix region, while weakly annotated sets have only the bounding box around the cervix region. Mask R-CNN can only be trained using strongly annotated data; however, Mask^X^ R-CNN can be trained using both dataset types. For example, we train a Mask^X^ R-CNN model on a combination of weakly annotated data and strongly annotated data that was also used to train the Mask R-CNN model. We compare the segmentation results using five strategies: (1) simple single- and cross-dataset training and testing: training and testing data is sourced from only one dataset, but the dataset used for training or testing could be different; (2) boosting with bounding box information: evaluate segmentation performance when adding weakly annotated images to a strongly annotated training set; (3) modified weak annotations: where bounding boxes are relaxed to include neighboring pixels to allow uncertainty in expert observer annotation; (4) multi-dataset training and testing: training and/or test data are pooled from multiple datasets; (5) different training strategies: where training is done end-to-end or stage-wise; and, both Mask R-CNN and Mask^X^ R-CNN, described below, are built on an underlying “backbone” network (e.g., ResNet50, ResNet101 [30]) that we compare results across.

### 4.1. Mask R-CNN

**Figure 3:**
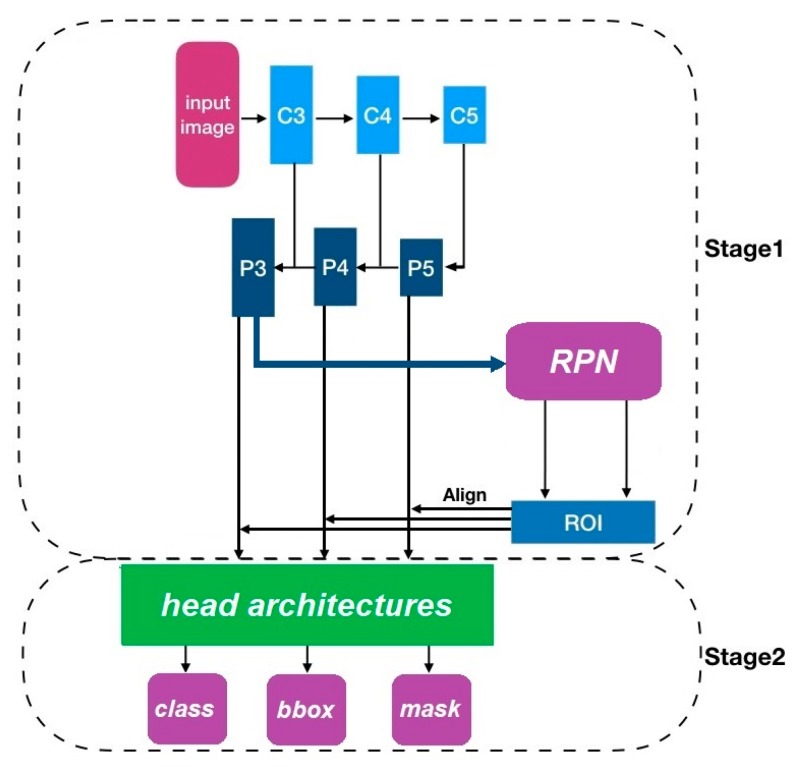
Schematic of Mask R-convolutional neural network (CNN) method. The features computed from the C3…C5, P3…P5 pyramid structure are fed into the Region Proposal Network (RPN) and also into the Mask R-CNN classification step. ROI: region of interests.

Mask R-CNN [9] is an extension of the Faster R-CNN [31] deep learning architecture for segmentation of object “instances” in an image. It adopts the same two-stage procedure as Faster R-CNN, where the first stage is a Region Proposal Network with a Feature Pyramid Network [32] structure, followed by a second stage predicting the class and bounding box in parallel. In addition, a mask branch is added in the second stage which is used to predict binary masks of the objects, yielding finer spatial definition. A feature pyramid structure extracts features on different scales for both the region proposal and classification (Figure 3). This may aid in improving accuracy and speed compared with single-scale feature map structures. Furthermore, a novel image region of interest (RoI) alignment technique, RoIAlign [9], is applied to compute the exact values of the input features at four regularly sampled locations in each evenly split RoI bin. Bilinear interpolation is employed to avoid the information loss brought by simply pooling and quantizing. Since the supervised architecture requires training with strongly annotated image data, we focus on training and testing Mask R-CNN using single or multiple datasets where mask ground truth is available (i.e., $A_{mask},\;B_{mask},$ and $C_{mask}$).

### 4.2. Mask^X^ R-CNN

**Figure 4:**
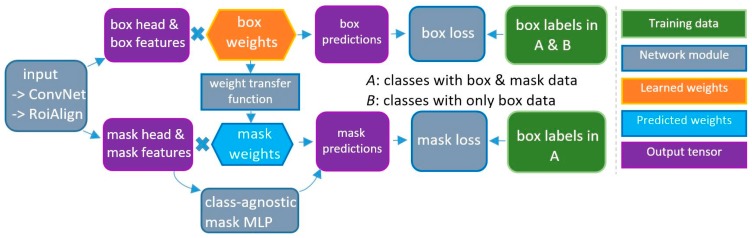
Detailed illustration of Mask^X^ R-CNN method (adapted from [10]).

Several other deep learning methods in the R-CNN family also require strongly annotated data, limiting their use when only object bounding boxes are available. The Mask^X^ R-CNN [10] network was created to address this “data annotation gap” in the training process by taking advantage of both types of annotations, bounding box and mask. Briefly, Mask^X^ R-CNN augments the Faster R-CNN bounding box prediction model with additional mask processing which consists of a small convolutional neural network (weight transfer network) and a Multi-Layer Perceptron (MLP), together known as “mask heads” (Figure 4). Mask^X^ R-CNN can be trained using two strategies, depending on how “mask heads” are used: (i) stage-wise training; and, (ii) end-to-end training.

#### 4.2.1. Stage-Wise Training

Since Mask^X^ R-CNN is built upon Faster R-CNN by adding mask heads; its training can be separated into two stages. In the first stage, a Faster R-CNN is trained using only the bounding box ground truth provided. In the second stage, the mask heads are trained while freezing the bounding box head and features. We provide only a small number of mask ground truth images in the second stage to help the mask heads learn to convert the bounding box prediction weights obtained in the first stage to fine mask boundary predictions. Taking our datasets A and B as an example, we train the network using training split of $A_{box}$ and $B_{box}$ in the first stage and then use the related $A_{mask}$ or $B_{mask}$ only in the second stage. Finally, we evaluate the network on the test splits of both A and B datasets. We also apply this training strategy over all possible combinations of our datasets A, B, and C.

#### 4.2.2. End-to-End Training

It was reported in both [9] and [10] that simultaneous multi-task training can achieve better performance than working with each task separately in stage-wise training for the COCO dataset. In our scenario, we also conduct end-to-end training which jointly trains the bounding box head and the mask heads. Note that the bounding box weight gradient is not computed to avoid the class-specific weight discrepancy problem as stated in [10]. We train the same dataset combinations in this manner as we do in stage-wise training; the comparison is discussed in later sections. This is an interesting study because the COCO dataset comprises images that are quite different in appearance and content, while our images are very similar overall, particularly in the cervix region.

### 4.3. Implementation Details

We resize images such that their shorter edge is 532 pixels and maintain the image aspect ratio. The training is done on high performance computers powered with Nvidia Tesla V100 graphical processing units (GPU). We use mini-batch size of 2 images per GPU and an RoI mini-batch size of 512. An RoI is considered acceptable if it has IoU with a ground-truth box of at least 0.5. We train the algorithm on 1 GPU for 180,000 iterations with a learning rate starting at 0.001 and decaying by 10 times at iterations 80,000 and 120,000. The weight decay factor and momentum is set to be 0.0001 and 0.9, respectively. We use ResNet50 and ResNet101 [30] separately as the backbone, with 1 image per GPU and the same number of iterations, initializing with ImageNet pre-trained weights. We apply only scaling augmentation, although it is possible that more data augmentation might help in improving the overall performance.

## 5. Evaluation and Discussion

We compare the segmentation results using the Dice and IoU scores calculated from the predicted segmentation masks. As mentioned above there are five strategies for evaluating the algorithms, viz. (1) simple single- and cross-dataset training and testing; (2) boosting with bounding box information; (3) modified weak annotation; (4) multi-dataset training and testing; and (5) different training strategies.

### 5.1. Single- and Cross-Dataset Training and Testing

We train Mask R-CNN with $A_{mask}$ and $C_{mask}$ separately and test each of the models on respective test subsets. We use the following 4 training and testing scenarios: (1) train with $A_{mask}$, test on test split of A; (2) train with $C_{mask}$, test on test split of C; (3) train with $A_{mask}$, test on test split of C; and, (4) train with $C_{mask}$, test on test split of A. By observing the results of steps (1) and (2) we can evaluate the performance and inspect the robustness of Mask R-CNN in fully supervised learning within the same dataset. Meanwhile, to understand the difference in performance across datasets, we compare the quantitative results of steps (1) and (3), as well as of (2) and (4), where the same model is tested on different datasets.

**Figure 5:**
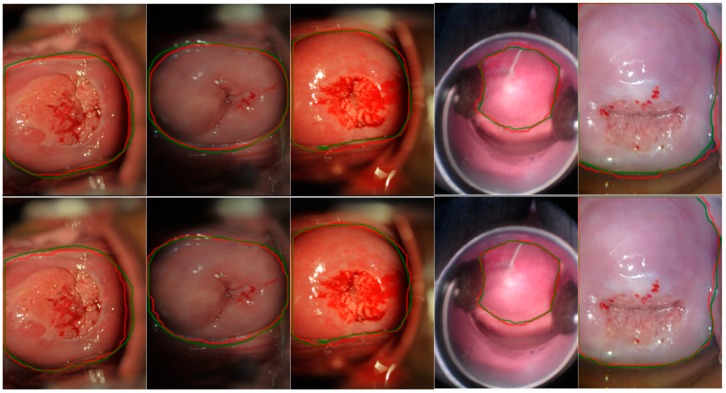
Visualization and Comparison of results from Mask R-CNN and Mask^X^ R-CNN tested on datasets A and C. The 1st row images are test results from Mask^X^ R-CNN, the 2nd row images are results from Mask R-CNN. The green lines are the ground truth marked by human experts, red lines are model predictions.

Also, for Mask^X^ R-CNN we apply the same 4 training scenarios with only slight differences. For example, for the stage-wise training of (1) above, we trained the first stage Faster R-CNN with $A_{\_box}$ and then we trained the second stage mask heads with $A_{mask}$. We tested the trained model on the test split within the same dataset, as well as across datasets. We further compared each of the 4 testing results of Mask^X^ R-CNN with the corresponding result obtained from Mask R-CNN, in order to (1) observe the performance difference between the two deep learning architectures on the same task; and, (2) as a verification on the correctness of test results from the two architectures. The segmentation results are visualized in Figure 5 with both ground truth and predicted contours marked, and the quantitative results are provided in Table 2.

From Table 2, we see that for training and testing with the same dataset: (i) Mask R-CNN achieves best (Dice, IoU) scores on dataset A of (0.9446, 0.8970) with ResNet50, as compared to the best scores on dataset C of (0.9197, 0.8673) achieved using ResNet101; and, (ii) Mask^X^ R-CNN network achieves best scores on dataset A of (0.9418, 0.8925) with ResNet101, as compared to best scores on dataset C of (0.8867, 0.8153) also using ResNet101. Note that we obtain higher scores for dataset A as compared to C for both network architectures. A possible interpretation is that dataset C images are inherently more complex for this task, or there are insufficient number of images for representing the variety in cervix appearance.

**Figure 6:**
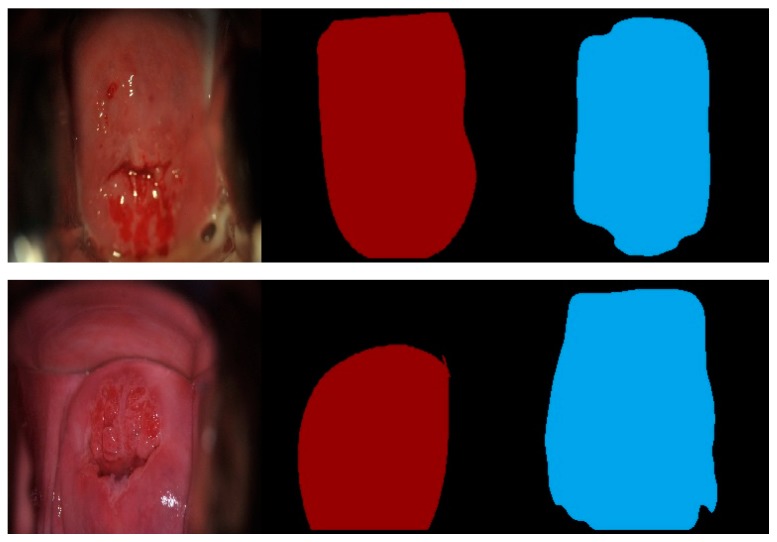
U-Net segmentation examples. Left: original image; middle (red): ground truth; right (blue): automatic segmentation. The top-row segmentation has an intersection-over-union (IoU) score of 0.834, the bottom-row segmentation has an IoU score of 0.671.

We have also investigated the widely used image object segmentation method, U-Net [32], and compared the U-net results with Mask R-CNN and Mask^X^ R-CNN in our study. Compared with the Mask R-CNN (IoU: 0.8970) and Mask^X^ R-CNN (IoU: 0.8925) presented above, U-Net achieved a significantly lower IoU (0.741). Note that the U-Net is trained using the train split of dataset A and tested on the test split of dataset A, and segmentation sample images are shown in Figure 6 below:

As shown in Table 2, we also conducted training and testing across datasets. When we train the model on A, test the model on C, Mask R-CNN achieves best (Dice, IoU) scores of (0.7578, 0.6456), and Mask^X^ R-CNN achieves (0.7798, 0.6646). Note that these scores are considerably lower than when dataset C was trained and tested on itself. When we reverse the training and testing roles, and train on C and test on A, Mask R-CNN achieves (0.9164, 0.8497), and Mask^X^ R-CNN achieves (0.8902, 0.8075) both using the ResNet101 backbone.

Observation: when training on dataset C the performance is approximately stable across network architectures (Mask R-CNN or Mask^X^ R-CNN) and across test datasets (A or C); when training on dataset A, performance is approximately stable across network architectures, but differs significantly across test datasets. Results in Table 3 show that our methods outperform the state of the art from Section 2.

Since dataset B should not be split into training and test subsets, we use the dataset here for testing only. As shown in Table 4, we obtain higher scores using the model trained with dataset C than the one trained with dataset A.

### 5.2. Boosting with Bounding Box Information and Mask^X^ R-CNN

As discussed in Section 5.1, the performance of cross-dataset testing may be significantly impacted when the training data is not sufficiently representative of the test data; this happens when we train with dataset A and test on dataset C. This negative impact may be expected to worsen when the quantity of strongly labeled data is very small. We investigate the effectiveness of Mask^X^ R-CNN for training when we have a large collection of weakly annotated data, but only a small collection of strongly annotated data. Specifically, we train Mask^X^ R-CNN with the (weakly annotated) $A_{box}$ and $C_{box}$ data pooled with the (strongly annotated) $A_{mask}$ data. Mask^X^ R-CNN may play a significant role in this case, by using both and $C_{box}$ for training the bounding box head and $A_{mask}$ for training the mask head. We carry out this procedure and compare this result with the one obtained in Section 5.1. Table 5 shows the Dice/IoU scores obtained.

**Figure 7:**
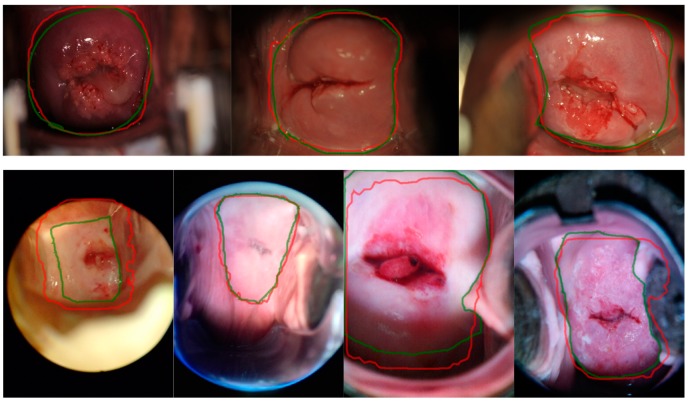
Segmentation examples of Mask^X^ R-CNN trained with A + $C_{\_box}$. Top row: images from dataset A. Bottom row: images from dataset C. The green line denotes the ground truth boundaries, the red line indicates the automatic boundaries.

Compared with training with only $A_{mask}$, as described in Section 5.1, and adding the additional $C_{box}$ data in training, the Mask^X^ R-CNN achieves (Dice, IoU) scores of (0.9418, 0.8922), using ResNet50, on dataset A, and (0.8993, 0.8220), using ResNet50, on dataset C. The performance on dataset A remains similar to the results of training and testing on dataset A reported in Table 2; this shows that the model is robust on dataset A with respect to the mixture of $A_{mask}$ and $C_{\mathrm{box}}$ for training. Furthermore, a large performance boost of (0.1195, 0.1574) in (Dice, IoU) up from previously obtained 0.7798, 0.6646, when training on A, testing on C can be observed, showing that the segmentation is improved by utilizing additional bounding box information in Mask^X^ R-CNN. Figure 7 shows the segmentation example of this model tested on datasets A and C.

### 5.3. Modified Weak Annotation

We notice variation among the cervix fine boundaries that are marked by different experts. This implies that the weak annotations might also vary. We evaluate the resilience of the segmentation algorithm to variations in bounding box size. For this, we create three datasets by relaxing $C_{box}$ annotation boundaries by 10, 20, and a random number of pixels in the range (0, 35). Then we train the first stage with these modified bounding box annotation sets. We use the same $A_{mask}$ in the second stage to train the mask heads. We compare the three corresponding results with those we presented in the previous section. Note that we are using images of approximate size 530 × 400 pixels, so any changes of magnitude 10–30 pixels could add significant noise to our training dataset and models.

As shown in Table 6, we find that when a bounding box is extended in size, the performance (Dice, IoU) of our model remains stable (0.9376, 0.8850) on the strongly annotated dataset A. As for dataset C, we get a Dice score of 0.8413 if the bounding boxes are extended by random numbers of pixels, and this is higher than the 20-pixel extension results and lower than the 10-pixel extension results. We can see that our models remain relatively stable and robust to the modifications on training dataset. Comparing the Dice and IoU scores between Table 5 and Table 6, we find that the performance on dataset C drops after extending the bounding boxes, and degrades further if we keep doing so. This is consistent with our expectations since dataset C only provides bounding box annotation and the testing performance should be highly correlated with the visual content within the bounding boxes.

### 5.4. Multi-Datasets Training and Testing

We further examine the segmentation performance on both Mask R-CNN and Mask^X^ R-CNN trained with more than one dataset, using seven (7) box and mask dataset combinations for training and testing. Firstly, we train the Mask R-CNN with combinations of $A_{mask}$, $B_{mask}$, and $C_{mask}$. We also train a Mask^X^ R-CNN with the same dataset but with the bounding box in the first stage and mask in the second stage. Secondly, we train Mask^X^ R-CNN only, utilizing combinations of mask and bounding box components of the three datasets. In each training dataset combination, we have at least one mask dataset and at most two bounding box datasets. Results are shown in Table 7 below.

Observations: overall, for Mask R-CNN, training with all the strongly annotated datasets, $A_{mask}$, $B_{mask}$, and $C_{mask}$ together gives the best performance. When substituting the weakly annotated dataset(s) for the corresponding strongly annotated datasets, we can still achieve comparable performance using Mask^X^ R-CNN.

### 5.5. Comparing Stage-Wise Training to End-to-End Training

Results presented so far for Mask^X^ R-CNN use stage-wise training, however, according to [10], this approach could be suboptimal. To evaluate this assertion, we evaluate end-to-end training for Mask^X^ R-CNN on datasets A and C and compare its performance with those obtained above. Table 8 shows the results which indicate the end-to-end training, indeed, improve segmentation performance.

As for timing, for stage-wise training and taking “A + C” as an example, training Mask^X^ R-CNN takes 5 h on ResNet50 and 6.5 h on ResNet101 for training two stages. On the other hand, end-to-end training takes 4 h on ResNet50 and 5.3 h on ResNet101.

### 5.6. Error Cases

**Figure 8:**
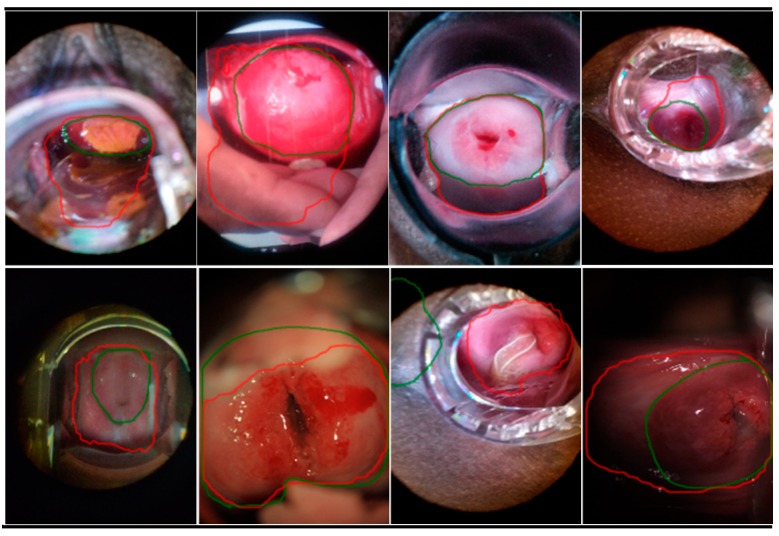
Tested images with Dice scores < 0.9. The green lines are the ground truth marked by human experts, red lines are model predictions.

We also study images that have relatively low Dice and IoU scores, which we denote as “error cases”. In Figure 8, we show examples of such cases (Dice score < 0.9) due to mismatch with pre-defined ground truth; most of these have the presence of visual distractors such as pubic hair, intra-uterine devices (IUDs), the speculum, and even parts of human hands. In some images we even find that the cervix region occupies less than 50% of the entire image. We also observe that low Dice/IoU scores could be a result of false negatives of the ground truth annotations, suggesting subjectivity in the markup.

## 6. Conclusions

In this work, we have presented an investigation of state-of-the-art deep learning methods for automatic cervix region segmentation and their resilience to variation in expert annotation and their robustness to diversity in appearance of cervix in the image. Toward this goal, we evaluated the algorithms on multiple strongly and weakly annotated datasets, both for homogeneous (“within dataset”) and heterogeneous (“across datasets”) data. We also explore the effect of training with a mix of a small fraction of strongly annotated data and a large fraction of modified weakly annotated data from a different dataset, thereby evaluating the reliability and robustness of the models to perturbations and variations in expert observer annotations.

From the results we obtained, we can see that dataset C is more challenging and, perhaps, representative of the real world variety in image acquisition. Further, we achieved our best performance when using all three datasets in training a Mask R-CNN. When substituting the weakly annotated dataset(s) for the corresponding strongly annotated datasets, we can still achieve comparable performance using Mask^X^ R-CNN. More importantly, we find that with Mask^X^ R-CNN, the cross-dataset performance can be significantly boosted with additional data having only bounding box annotations. This is a promising finding suggesting that using more weakly annotated image data can serve as a surrogate when fine-boundary segmentation is of insufficient quantity.

Accurate cervix segmentation can help in disease tracking and perhaps even assist in automated longitudinal analysis after biopsy or training an artificial intelligence (AI) algorithm to augment therapeutic decision-making. It can also assist in retrieving images with similar lesions for visual analysis in large datasets. As next steps, we plan to continue optimizing the algorithm with respect to segmentation performance.

## Figures and Tables

**Table 1:** Quantitative details of Datasets A, B, and C.

Dataset	Training Split	Testing Split
A	2797 (from 1354 women)	601 (from 320 women)
B	This dataset contains 939 images, which are used, either (1) all as training data; or (2) all as testing data. All the images have mask annotations and box annotations.	
C	1448	502

**Table 2:** Results for training within and across datasets (“BaseNet” denotes the basic deep learning structure used in feature pyramid and region proposal network). The **green bold font** highlights the compared results obtained in single dataset training and testing, and **red bold font** highlights the compared results obtained in cross-dataset training and testing.

Training Dataset	BaseNet	Testing Results on A			
		Mask R-CNN		Mask^X^ R-CNN	
		Dice	IoU	Dice	IoU
A	ResNet50	**0.9446**	**0.8970**	0.9405	0.8902
	ResNet101	0.9438	0.8960	**0.9418**	**0.8925**
C	ResNet50	0.9107	0.8401	0.8664	0.7711
	ResNet101	**0.9164**	**0.8497**	**0.8902**	**0.8075**
		**Testing Results on C**			
A	ResNet50	0.7385	0.6296	**0.7798**	**0.6646**
	ResNet101	**0.7578**	**0.6456**	0.7704	0.6547
C	ResNet50	0.9106	0.8572	0.8788	0.8058
	ResNet101	**0.9197**	**0.8673**	**0.8867**	**0.8153**

**Table 3:** Baseline performance for cervical region segmentation from previously reported studies.

Ref.	Images	Measurements
[11]	110 from 100 women	IoU: 0.79
[12]	NA	IoU: 0.76
[13]	151	Dice: 0.90
[14]	278 (subset of Dataset B)	Dice: 0.75
[16]	378 (subset of Dataset B)	Dice: 0.81
[17]	250 from 4 women	AUC: 0.90
[18,19,20]	100	sensitivity: 0.71 specificity: 0.82
[22]	Dataset A	IoU: 0.74
[23,24,25]	1480 (Dataset C)	Dice: 0.67
[26]	Dataset A	(Dice, IoU): (0.8711, 0.765)

**Table 4:** Results for training on dataset A or C and testing on dataset B.

Training Dataset	BaseNet	Testing Results on B			
		Mask R-CNN		Mask^X^ R-CNN	
		Dice	IoU	Dice	IoU
A	ResNet50	0.8443	0.7522	0.8345	0.7377
	ResNet101	0.8481	0.7569	0.8359	0.7395
C	ResNet50	0.8863	0.8166	0.8700	0.7904
	ResNet101	0.8873	0.8186	0.8747	0.7976

**Table 5:** Mask^X^ R-CNN DIC/IoU scores obtained with datasets A and $C_{\_box}$ .

Training Dataset	BaseNet	Testing Results on A		Testing Results on C	
		Dice	IoU	Dice	IoU
A + $C_{\_box}$	ResNet50	0.9418	0.8922	0.8993	0.8220
	ResNet101	0.9410	0.8910	0.8910	0.8093

**Table 6:** Quantitative results for stage-wise training (ResNet50) using dynamic bounding box sizes.

Training Dataset	Dynamic Modifier	Testing Results on A		Testing Results on C	
		Dice	IoU	Dice	IoU
A + $C_{\_box}$	10 pixels	0.9376	0.8850	0.8635	0.7670
	20 pixels	0.9369	0.8829	0.8280	0.7164
	Rand (0, 35) pixels	0.9387	0.8863	0.8413	0.7350

**Table 7:** Results when training with multiple datasets. The “(box)” denotes that only the bounding box annotations of this dataset are used in training; otherwise, mask annotations are used. Best results are highlighted in bold font.

Training Dataset	Testing Dataset	BaseNet	Mask R-CNN		Mask^X^ R-CNN	
			Dice	IoU	Dice	IoU
A + C	A	ResNet50	0.9446	0.8971	0.9138	0.8458
		ResNet101	**0.9469**	**0.9009**	0.9231	0.8622
	C	ResNet50	0.9161	0.8630	0.8866	0.8073
		ResNet101	0.9200	0.8694	0.8758	0.7908
A + B	A	ResNet50	0.9442	0.8966	0.9437	0.8956
		ResNet101	0.9442	0.8964	0.9421	0.8932
	C	ResNet50	0.8176	0.7230	0.8158	0.7159
		ResNet101	0.8219	0.7216	0.8066	0.7017
A + B + C	A	ResNet50	0.9454	0.8985	0.9376	0.8852
		ResNet101	0.9462	0.8999	0.9325	0.8713
	C	ResNet50	0.9159	0.8623	0.8985	0.8228
		ResNet101	0.9213	0.8700	0.8904	0.8010
A + B + C(box)	A	ResNet50	N/A	N/A	0.9387	0.8870
		ResNet101	N/A	N/A	0.9376	0.8857
	C	ResNet50	N/A	N/A	0.8915	0.8102
		ResNet101	N/A	N/A	0.8866	0.8021
A(box) + B + C(box)	A	ResNet50	N/A	N/A	0.9244	0.8623
		ResNet101	N/A	N/A	0.9050	0.8311
	C	ResNet50	N/A	N/A	0.8841	0.7966
		ResNet101	N/A	N/A	0.8819	0.7945
A(box) + B + C	A	ResNet50	N/A	N/A	0.9318	0.8751
		ResNet101	N/A	N/A	0.9353	0.8809
	C	ResNet50	N/A	N/A	0.8962	0.8190
		ResNet101	N/A	N/A	0.8869	0.8038
A(box) + B(box) + C	A	ResNet50	N/A	N/A	0.9294	0.8710
		ResNet101	N/A	N/A	0.9308	0.8732
	C	ResNet50	N/A	N/A	0.8928	0.8139
		ResNet101	N/A	N/A	0.8906	0.8096

**Table 8:** Performance comparison between stage-wise training and end-to-end training strategies, quantitative results obtained by testing datasets A and C using ResNet50 as BaseNet.

Training Dataset	Training Strategy	Mask^X^ R-CNN			
		Testing Results on A		Testing Results on C	
		Dice	IoU	Dice	IoU
A + C	Stage-wise	0.9138	0.8458	0.8866	0.8073
	End-to-end	0.9452	0.8980	0.9081	0.8368
